# Genotyping-by-Sequencing Based Investigation of Population Structure and Genome Wide Association Studies for Seven Agronomically Important Traits in a Set of 346 *Oryza rufipogon* Accessions

**DOI:** 10.1186/s12284-022-00582-4

**Published:** 2022-07-12

**Authors:** Palvi Malik, Mao Huang, Kumari Neelam, Dharminder Bhatia, Ramanjeet Kaur, Bharat Yadav, Jasdeep Singh, Clay Sneller, Kuldeep Singh

**Affiliations:** 1 School of Agricultural Biotechnology, Punjab Agricultural University, Ludhiana, India; 2 Department of Horticulture and Crop Science, OARDC, The Ohio State University, Wooster, USA; 3 Department of Plant Breeding and Genetics, Punjab Agricultural University, Ludhiana, India; 4 Crop Pathology and Genetics Lab, University of British Columbia, Vancouver, Canada; 5 International Crops Research Institute for the Semi-Arid Tropics (ICRISAT), Hyderabad, Telangana India

**Keywords:** *Oryza rufipogon*, Population structure, Productivity related traits, SNP tagging, Genome-wide association study, Minimum Bayes Factor, LD decay, Gene annotation

## Abstract

Being one of the most important staple dietary constituents globally, genetic enhancement of cultivated rice for yield, agronomically important traits is of substantial importance. Even though the climatic factors and crop management practices impact complex traits like yield immensely, the contribution of variation by underlying genetic factors surpasses them all. Previous studies have highlighted the importance of utilizing exotic germplasm, landraces in enhancing the diversity of gene pool, leading to better selections and thus superior cultivars. Thus, to fully exploit the potential of progenitor of Asian cultivated rice for productivity related traits, genome wide association study (GWAS) for seven agronomically important traits was conducted on a panel of 346 *O. rufipogon* accessions using a set of 15,083 high-quality single nucleotide polymorphic markers. The phenotypic data analysis indicated large continuous variation for all the traits under study, with a significant negative correlation observed between grain parameters and agronomic parameters like plant height, culm thickness. The presence of 74.28% admixtures in the panel as revealed by investigating population structure indicated the panel to be very poorly genetically differentiated, with rapid LD decay. The genome-wide association analyses revealed a total of 47 strong MTAs with 19 SNPs located in/close to previously reported QTL/genic regions providing a positive analytic proof for our studies. The allelic differences of significant MTAs were found to be statistically significant at 34 genomic regions. A total of 51 *O. rufipogon* accessions harboured combination of superior alleles and thus serve as potential candidates for accelerating rice breeding programs. The present study identified 27 novel SNPs to be significantly associated with different traits. Allelic differences between cultivated and wild rice at significant MTAs determined superior alleles to be absent at 12 positions implying substantial scope of improvement by their targeted introgression into cultivars. Introgression of novel significant genomic regions into breeder’s pool would broaden the genetic base of cultivated rice, thus making the crop more resilient.

**Supplementary Information:**

The online version contains supplementary material available at 10.1186/s12284-022-00582-4.

## Background

To feed nearly 10 billion people by 2050, agricultural production must be increased by 60% from 2005 base year (Alexandratos 2012). The global annual yield increase in rice during the first decade of the current century has been < 1.0% (Phillips 2010; Ray et al. 2013), and the fact that agriculture is experiencing greater competition for land, water, and energy makes it sceptical whether the requisite growth rate could be achieved. Considering the erratic climatic changes along with challenges posed by abiotic and biotic stresses, increasing the rice productivity without increasing land under cultivation is a big challenge for rice breeders (Foley et al. 2011; Qian et al. 2016; Zeng et al. 2017). Compounding the problem is the current practice of crossing elite lines, which is expected to reduce genetic variability in the working germplasm, thus, preventing the discovery of novel traits to improve yield. Undoubtedly, plant breeders have witnessed a substantial increase in yield over the years with adoption of new cultivars and better management practices (Sanchez et al. 2013). But, in order to solve the envisioned 9 billion people question (Jacquemin et al. 2013), the rate of rice production must increase on the currently available land.

The Asian cultivated rice, *O. sativa*, belongs to genus *Oryza* that includes another cultivated African rice species, *O. glabberima* (2*n* = 24, AA) and 22 wild species (2*n* = 24, 48) representing the AA, BB, CC, BBCC, CCDD, EE, FF, GG, KKLL, and HHJJ genome types (Sanchez et al. 2013). It has been envisioned that utilizing the useful novel variability present in wild relatives of rice could be a promising approach to increase the genetic variability in a breeder’s pool. The wild relatives are an important genetic resource for breeding and genomics research as they are a reservoir of useful genes/QTLs for tolerance to major abiotic and biotic stresses, yield-related traits including weed-competitive ability, new source of cytoplasmic male sterility, and other traits related to rice improvement (Brar and Khush 2018). Of the several approaches advocated to further improve rice productivity, utilization of wild species is of substantial importance (Khush 2005, 2013).

A large amount of untapped genetic variations and higher percentage of fertile hybrids obtained from inter specific crosses of *O. sativa* with ancestral species, *O. rufipogon* has made the progenitor an attractive choice for rice breeders. It has been utilized not only for improving qualitative and quantitative traits but also for introgressing new useful variability which recognizes its potential as a valuable reservoir of genetic variation (Tanksley and McCouch 1997; Brar and Khush 2018; Dalmacio et al. 2005). Different kinds of populations such as advanced backcross populations, backcross inbred lines, chromosome segment substitution lines, near-isogenic lines, and recombinant inbred lines have been derived from crosses between *O. rufipogon* and *O. sativa* as a pre-breeding material (Neelam et al. 2018). Genes for biotic stress like bacterial blight resistance (Zhang et al. 1998; Utami et al. 2008), brown planthopper resistance (Deen et al. 2017), tungro virus tolerance (Kobayashi et al. 1993), and abiotic stress tolerance like acidic conditions, iron toxicity, phosphorus deficiency have been transferred from *O*. *rufipogon* into rice cultivars by McCouch et al. (2007) and Brar and Khush (2006). Similarly, there have been a number of studies where introgression lines and back-cross populations derived from *O. rufipogon* accessions have been used to map yield related QTLs. Moncada et al. (2001) identified three QTLs for grain number, *gpl.1*, *gpl2.1*, *gpl11.1* in back-cross population utilizing AB-QTL approach. Marri et al. (2005) mapped 3 QTLs each for grain number (*gnp2.1*, *gnp2.2*, *gnp5.1*), spikelet number per panicle (*snp2.1*, *snp5.1*, *snp5.2*), yield (*yldp2.1*, *yldp2.2*, *yldp9.1*) and four QTLs for thousand grain weight (*gy2.1*, *gy2.2*, *gy2.3*, *gy9.2*) in BC_2_F_1_ population derived from *O. rufipogon IC22015*. Septiningsih et al. (2003) evaluated performance of 400 BC_2_F_2_ families derived from *O. rufipogon* accession IRGC105491 for mapping yield and yield components. The study reported three QTLs each for grain size (*gw1.1*, *gw3.1*, *gw3.2*), spikelet number per panicle (*spp2.1*, *spp3.1*, *spp9.1*), yield (*yld1.1*, *yld1.2*, *yld2.1*) and 1 QTL, *gpl1.1*, for grain number. Likely, Xiao et al. (1998) identified 6 QTLs (*gpl1.1*, *gpl2.1*, *gpl4.1*, *gpl5.1*, *gpl8.1*, *gpl8.2*) for grain number in BC_2_ population derived from IRGC 105,491. Fu et al. (2010) identified a total of 26 QTLs related to grain number, thousand grain weight and yield in BC_2_F_2_ and BC_2_F_4_ populations derived from Yuanjiang *Oryza rufipogon* Griff. In addition, Xie et al. (2008) and Jin et al. (2009) mapped grain number QTLs *gn9.1* and on *gpp8* chromosome 9 and 8 in BC_3_F_4_ and F_2:3_ populations, based on *O. rufipogon* accession IRGC105491. Xie et al. (2006) mapped a grain size locus, *gw8.1*, on chromosome 8 in BC_3_F_3_ population. Similarly, Liu et al. (2009) mapped two QTLs, *qspp1*, *qspp11* and Luo et al. (2013) mapped *qSPP5*, respectively, for spikelet number per panicle in ILs and BC_5_F_4_ population. Gaikwad et al 2014 mapped *spp1*, *gpp1*, *yld1* QTLs for spikelets per panicle, grains per panicle and grain yield in introgression lines derived from *O. rufipogon* accession IRGC100219. BILs derived from *O. rufipogon* accession IRGC104433 were used for mapping QTLs for thousand grain weight, grain weight and grain length and were designated as *qtgw5.1*, *qgw5.1* and *qgl7.1 *(Bhatia et al 2018). Thus, the previous studies have utilized only a few accessions of *O. rufipogon*. Similarly, several yield enhancing loci like *yld1.1, yld1.2, yld2.1, yldp2.1, yldp2.2, yldp9.1* and yield-enhancing traits such as spikelet number, grain number, grain size, grain weight, and panicle length have been identified and mapped in populations developed from crosses of *O*. *sativa* × *O*. *rufipogon*. The results from various studies focused on enhancing yield support transgressive segregation for yield and related components, making *O. rufipogon* ideal germplasm for mining yield enhancing loci (McCouch et al. 2007). Similarly, several yield enhancing loci like *yld1.1, yld1.2, yld2.1, yldp2.1, yldp2.2, yldp9.1* and yield-enhancing traits such as spikelet number, grain number, grain size, grain weight, and panicle length have been identified and mapped in populations developed from crosses of *O*. *sativa* × *O*. *rufipogon*. The results from various studies focused on enhancing yield support transgressive segregation for yield and related components, making *O. rufipogon* ideal germplasm for mining yield enhancing loci (McCouch et al. 2007). Hence, in order to fully exploit the potential of wild germplasm, the present study was designed so as to comprehensively analyse a large panel of *O. rufipogon* accessions utilizing the technique of GWAS. This study has helped us to identify founder *O. rufipogon* lines that can be used to generate allelic diversity in cultivated germplasm.

Majority of the research on *O. rufipogon* has utilized only a few accessions in different biparental crosses, thus limiting the allelic diversity and genetic resolution. Genome-wide association studies has been extensively employed in order to overcome these limitations as it involves a large association mapping panel, thereby increasing the allelic diversity and mapping resolution. Also, it provides an estimation of the effects of various alleles on the target trait. Since GWAS exploits historic recombination, it helps in dissecting the molecular basis of traits at a finer resolution which increases its chance for immediate utility in breeding programs. With the advent of NGS based SNP markers, a high density of markers is tested for their association with the target traits, thus giving better resolution than biparental linkage mapping carried out with limited number of SSR markers. Given these advantages of GWAS over traditional bi-parental mapping, GWAS has established itself as a promising approach to dissect complex polygenic traits at allelic level in biological sciences. The present study was designed with an aim to exploit a diverse set of 346 *O. rufipogon* accessions for exploiting variation for seven agronomically important traits that affect yield directly or indirectly.

## Results

### Variation of Seven Agronomic Traits in Panel of *O. rufipogon* Accessions

**Fig. 1:**
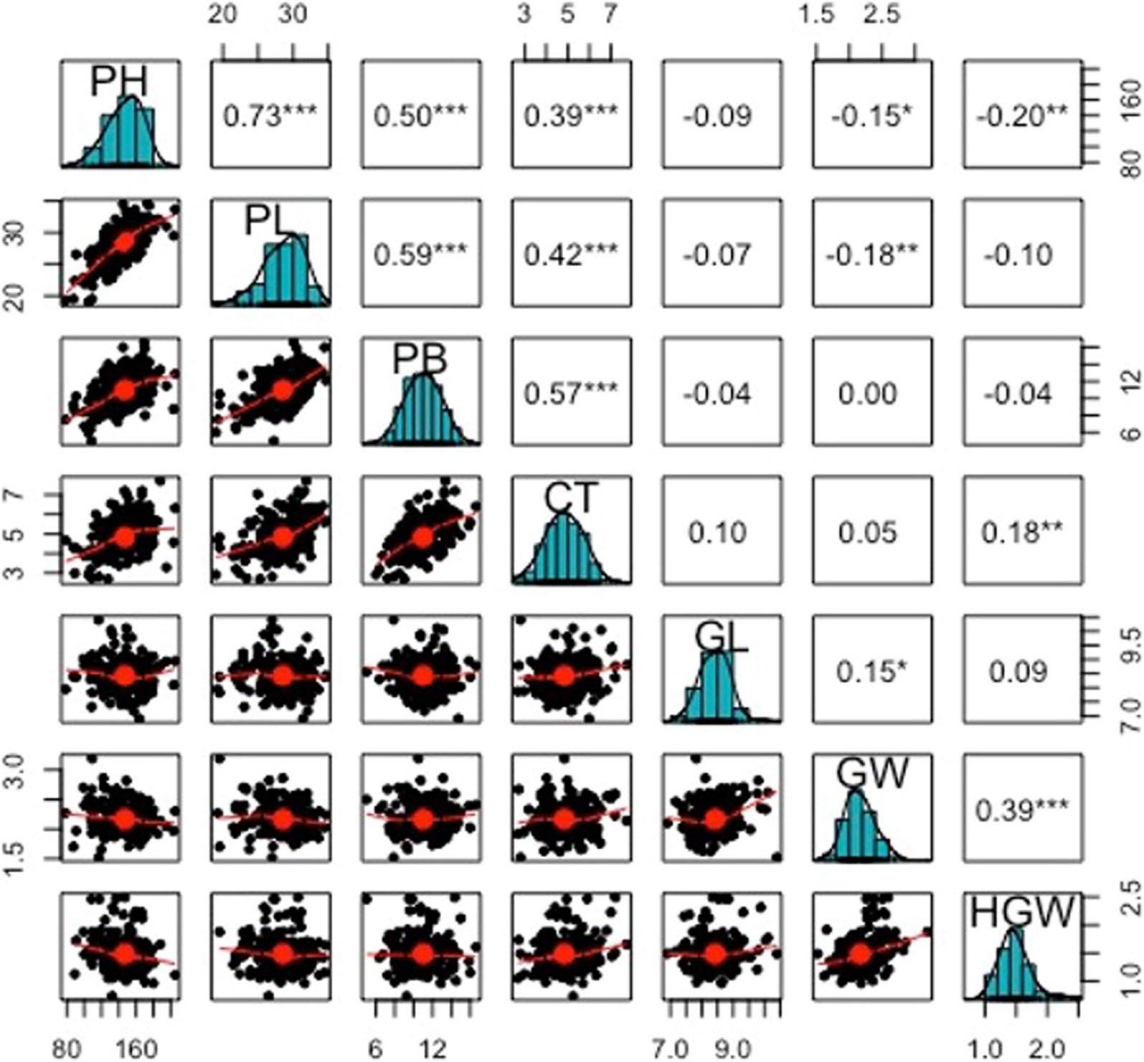
Correlogram showing the distribution of phenotypic data for various traits and pairwise correlations between various traits. PH, PL and PB showed a negative correlation with GL, GW and HGW

A large amount of variation for all the seven agronomic traits was recorded in *O. rufipogon* accessions. The frequency distribution curves of all the seven traits PH, CT, PL, PB, GL, GW and HGW revealed continuous variation for all the traits (Fig. 1). Pairwise correlations showed a negative trend of PH, PL and PB with all the grain parameters. The descriptive statistics and heritability measurements of the phenotypic traits are given in Table 1. Heritability ranged from 0.38 to 0.80 with minimum observed for panicle length and maximum for grain weight. A few accessions like IR104777, IR81989, IR100678, IR81802, IR93119 and IR104873 from Thailand, Myanmar, Taiwan, Indonesia, Cambodia and Thailand, respectively, were found to be better in terms of grain length and grain width. Similarly, a few Thailand accessions seem to be promising for promoting CT like IR104796, IR104775 and IR104792. Some other Thailand accessions, IR104783 and IR104766, had higher values of grain weight. Likewise, a Cambodian accession, IR110406, was recognized to have superior panicle architecture. Thus, many accessions were found to have the potential to be used in breeding systems to introduce beneficial genetic diversity into cultivated germplasm.

**Table 1:** Basic statistical summary of phenotypic data

Trait	Range	Mean	Heritability
PH	78.5–205.2 cm	147	0.59
CT	2.64–7.72 mm	4.83	0.57
PL	19.00–34.62 cm	28.54	0.38
PB	7.00–21	11.06	0.57
GL	6.9–10.4 mm	8.43	0.45
GW	1.52–3.19 mm	2.17	0.4
HGW	0.73–2.50 g	1.49	0.8

### Population Structure Analysis

**Fig. 2:**
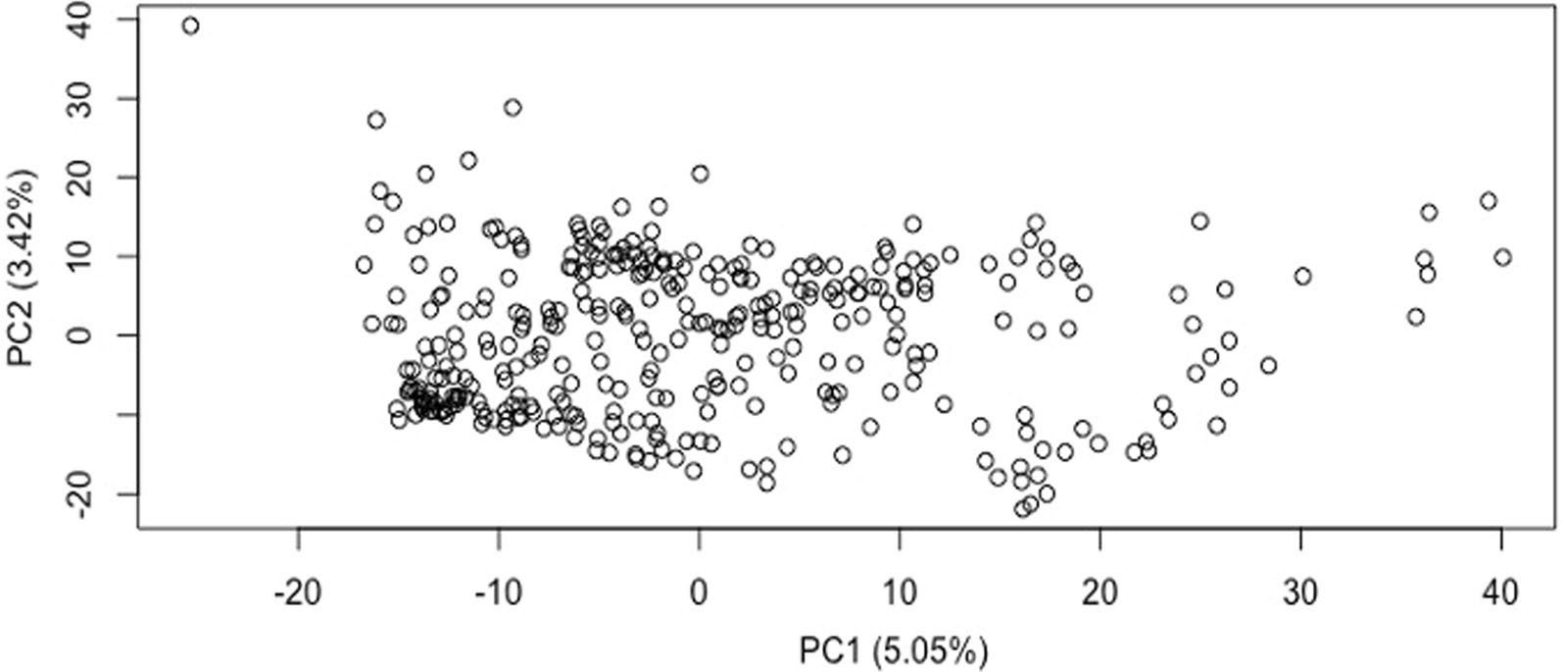
PCA plot generated from marker data of 346 accessions of *O. rufipogon* population from 16 different countries

**Fig. 3:**
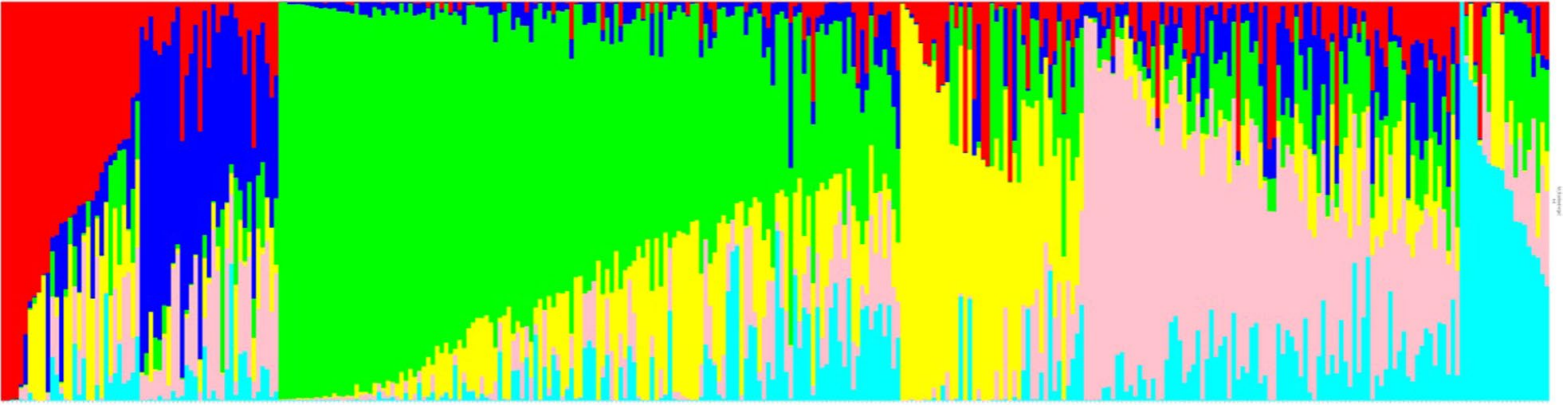
Classification of *O. rufipogon* population into six sub-populations represented by different colors: red, blue, green, yellow, pink, cyan

PCA plot didn’t reveal any distinct sub-grouping indicating absence of strong structure in the population (Fig. 2). Lack of clustering implies natural selection to have occurred in a continuous manner, leading to continuous diversity. Although bayesian model-based clustering by StrAuto suggested probable division into six-subpopulations but the level of differentiation was determined to be too low to call them genetically differentiated (Fig. 3). Considering the membership criterion of 75%, only eighty-nine accessions were classified into discrete sub-populations, and the remaining 257 were judged as admixed. Such a high proportion of admixtures led to blurring of the boundaries among different sub-populations, making this germplasm set an ideal panel for GWAS. High degree of admixture suggests a high degree of gene movement to have occurred between regions. Only a little correlation was observed between geographic coordinates and sub-populations.

Global *F*_*st*_ value of 0.06 denoted very low level of genetic differentiation, indicating only 6% of the total genetic variation to be distributed among subpopulations, and remaining 94% of the variation was present within subpopulations. However, *F*_*st*_ values showed a marked increase to 0.28, after removal of admixtures. AMOVA test (Table 2) further confirmed the results as only 10.74% of total marker variation was attributed between sub-populations and the remaining 89.26% of variation was observed within sub-populations. This also serves as evidence of presence of continuous variation and absence of discrete classification into sub-populations. By removing the admixtures, the marker variance between sub-populations increased to 30% instead of 10% and remaining 70% was observed within the sub-populations.

**Table 2:** AMOVA summary partitioning marker variance into within and between six sub-populations in complete set of *O. rufipogon* population

	Degrees of freedom	Sum of squares	Mean sum of squares	Sigma	Percent
Variation between sub-populations	5	37,691.01	7538.2	125.62	10.74
Variations within populations	340	354,885.5	1043.78	1043.78	89.26
Total variations	345	392,576.5	1137.9	1169.4	100

Based on PCA, STRUCTURE, *Fst*, AMOVA, the current analysis indicated a very weakly differentiated population, where admixed lines made up most of the population. The real structure of the population was masked by the presence of a large number of admixtures as removal of admixtures from the population enhanced *F*_*st*_, pairwise *F*_*st*_ values. Also, before performing GWAS, model-based selection suggested the highest BIC value when no PCs were used in the model as covariates. Therefore, in the current analysis, covariates obtained from studying population structure were not added to the GWAS model. Also, the LD decayed to its half maximum at less than 10 Kb.

### Genome Wide Association Study

Genome wide association study conducted on a set of 346 *O. rufipogon* accessions using tagged set of 15,083 SNP markers, revealed a total of 47 significant marker trait associations (MTAs) at *p*-value ≤ 1e-4 (Table 3). Deciding an appropriate threshold value for determining the significance of association of a genomic region with the trait under study is an important aspect in interpreting GWAS findings. In the current study, Bonferroni corrected *p* value, LD-based threshold came out to be 3.31499E-06 and 1.28205E−06, respectively. However, mBF based on Bayesian methods was calculated to be 0.00173379. A total of 10, 6 and 194 significant MTAs were obtained by using the Bonferroni, Ld based and mBF based corrections. However, for current study, *p*-value threshold was kept to be 1e-4 in order to keep a manageable number of significant SNPs for further in-depth annotation. The details of loci harbouring the significant SNPs or loci in the LD region of significant SNPs along with their functional annotation is given in Table 4. Of the 47 significant MTAs observed, 19 SNPs located in/close to previously reported QTL/genic regions such as *bct2b*, *bct11c*, *pl2a*, *qPL-3–2*, *qPL-6*, *qPRB-4a*, *qGL-6*, *qGW-1*, *qTGW1-2*, *gw1*, *gw4*, *gw5*, *gw11.1*, *AQDZ008, AQDZ009, AQCU085, AQCU149, QKw2b*, *AQEO021,* providing an analytic proof of the concept of our study (Table 3).

**Table 3:** MTAs identified through GWAS for seven productivity related traits in AM panel of 346 *O. rufipogon* accessions

SNP	Primary trait (*p*-value, effect)	Secondary trait (*p*-value, effect)	Previously reported QTLs/genes
S1_1931325	CT(9.20E−05, 3.5%), HGW (2.24E−05, 3.42%)		*gw1* (Yu et al. 1997)
S1_35199996	CT (4.86E−05, 6.17%)	PH (0.011, 6.80%)	
S2_21858276	CT (5.95E−06, 4.12%)	PB ( 4.98E−02, 3.19%)	*AQFP026/bct2b* (Mu et al. 2004)
S3_15,462,193	CT (1.70E−06, 6.75%)	PH (0.026303753, 5.56%), PB (0.021535861, 6.26%)	
S6_16,241,331	CT (7.80E−05, 6.44%)		*AQDZ008* (Kashiwagi and Ishimaru 2004)
S7_1,592,572	CT (8.89 E−05, 3.73%)	PB ( 2.20792E−4, 5.48%)	
S7_18,874,511	CT (2.79E−05, 7.83%)		*AQDZ009* (Kashiwagi and Ishimaru 2004)
S7_24,536,919	CT (2.99E−05, 7.27%)	PH (8.47 E−03, 13.23%)	
S9_21,545,679	CT (8.02E−06, 3.98%)		
S11_21,499,010	CT (9.40E−05, 3.56%)	PB (1.58 E−04, 5.46%), PH (1.88E−02, 3.18%)	*bct11c* (Mu et al. 2004)
S2_30,762,305	PL (5.91E−05, 2.13%)	PH (0.021162648, 3.20%)	*pl2a/AQDQ013* (Zhuang et al. 1997)
S3_32,359,666	PL (1.17E−05, 4.39%)		*qPL-3–2/CQX12* (Yamamoto et al. 2016)
S4_34,899,389	PL (5.89E−05, 4.06)		
S6_6,597,022	PL (1.37E−07, 4.70%)	PH(4.67879E−04, 7.92%), PB(0.005270489, 7%)	*qPL-6*/*AQGM011*, *AQDY083, AQDY082*, *AQCU085* (Suh et al. 2005; Kobayashi et al. 2003; Mei et al. 2003)
S6_11,174,827	PL(3.76E−06, 3.4%)	CT( 0.020179035, 3.17%), GW(0.026049429, 1.61%)	*AQCU085* (Mei et al. 2003)
S8_11,774,122	PL(4.83E−07, 8.34%)	PB(0.035139693, 10.31%)	*AQCU085* (Mei et al. 2003)
S9_4,933,781	PL(4.62E−05, 2.08%)		*AQCU149* (Mei et al. 2003)
S10_14,320,467	PL(3.59E−07, 3.1%)	PH(0.002110037, 4.37%)	
S10_16,385,834	PL(2.00E−06, 5.40%)	PH(0.030803675, 6.90%), PB(0.00164866, 10.75%)	
S11_7,438,223	PL(3.00E−05, 2.08%)		
S4_30,721,851	PB(7.49E−05, 5.56%)		*qPRB-4a* (Teng et al. 2002)
S7_24,282,724	PB(5.57E−05, 7.60%)	PH(0.042017316, 3.63%)	
S6_24,807,445	GL(6.01E−05, 1.89%)		*qGL-6* (Li et al. 2003)
S8_5,775,398	GL(9.86E−05, 1.84%)		
S1_40,142,074	GW(7.92E−05, 2.67%)		*qGW-1* (Wan et al. 2005)
S2_7,048,091	GW(6.96E−05, 2.25%)		
S2_22,216,515	GW(1.62E−05, 2.75%)		
S3_7,917,671	GW(5.35E−05, 4.64%)		
S4_12,374,542	GW(3.99E−06, 4.88%)		
S4_34,598,600	GW(8.93E−05, 3.35%)		
S5_19,130,617	GW(7.87E−06, 3.87%)		
S5_23,720,696	GW(4.31E−05, 2.90%)		
S5_28,157,471	GW(5.68E−06, 2.44%)	PH(0.002417007, 3.94%), HGW(0.002092217, 2.68%)	
S8_20,423,775	GW(9.81E−06, 3.00%)		
S8_24,621,885	GW(3.61E−06, 3.92%)	PB(0.022511555, 5.8%), PH(0.020274467, 5.36%)	
S10_111,061	GW(5.03E−06, 4.29%)		
S10_19,109,511	GW(1.47E−05, 2.4%)		
S10_19,238,621	GW(2.16E−05, 3.91%)		
S1_38,370,584	HGW(6.88E−07, 7.38%)		*gw1.2, qTGW1-2*, *gw1.1* (Moncada et al. 2001; Hittalmani et al. 2002; Septiningsih et al. 2003)
S2_2,875,772	HGW(6.46E−05, 3.49%)	GL(0.035508538, 0.98%), PH(0.001764352, 3.4%)	*QKw2b* (Li et al. 1997)
S2_3,873,759	HGW(1.55E−05, 6.38%)	GL(0.005263071, 2.61%)	*QKw2b* (Li et al. 1997)
S4_4,499,266	HGW(1.34E−10, 9.46%)		
S4_26,914,103	HGW(9.69E−07, 6.17%)		*AQE053*, *gw4* (Xiao et al. 1998; Brondani et al. 2002)
S4_31,316,844	HGW(5.89E−06, 5.43%)		*AQEO021* (Redoña and Mackill 1998)
S4_35,115,087	HGW(3.76E−05, 6.58%)	CT(0.002310095, 6.00%)	
S5_24,316,574	HGW(7.99E−08, 8.85%)		*gw5b*, *gw5*(Xiao et al 1998; Hua et al. 2002)
S11_19,062,952	HGW(5.30E−07, 8.05%)		*gw11.1* (Moncada et al. 2001)

**Table 4:** Functional annotation of loci harboring significant SNPs/ present in LD-region of significant SNPs

SNP	Loci harboring significant SNP/ in LD-region	Functions
S1_1931325	LOC_Os01g04330, LOC_Os01g04340	OsCML16—Calmodulin-related calcium sensor protein, expressed; hsp20/alpha crystallin family protein, putative, expressed
S1_35199996	LOC_Os01g60870	Expressed protein
S2_21858276	LOC_Os02g36220	Terpene synthase, putative, expressed
S3_15,462,193	LOC_Os03g27003, LOC_Os02g36220	Expressed protein
S6_16,241,331	LOC_Os06g28550	nmrA-like family domain containing protein
S7_1,592,572	LOC_Os07g03810	Lectin-like receptor kinase 7
S7_18,874,511	LOC_Os07g31770	Chalcone synthase gene
S7_24,536,919	LOC_Os07g41014	Glycosyl hydrolases family 17 protein
S9_21,545,679	LOC_Os09g37280	Peroxisomal multifunctional enzyme type 2 protein
S11_21,499,010	LOC_Os11g36460, LOC_Os11g36470	SMC-related protein MSS2; ubiquitin carboxyl-terminal hydrolase 21
S2_30,762,305	LOC_Os02g50370	Helicase domain-containing protein
S3_32,359,666	LOC_Os03g56784	Expressed protein
S4_34,899,389	LOC_Os04g58690, LOC_Os04g58700	tRNA-specific adenosine deaminase 1; expressed protein
S6_6,597,022	LOC_Os06g12260, LOC_Os06g12250, LOC_Os06g12280	N-rich protein; sphingolipid C4-hydroxylase SUR2; glycosyl transferase 8 domain containing protein
S6_11,174,827	LOC_Os06g19590, LOC_Os06g19600, LOC_Os06g19610	Estradiol 17-beta-dehydrogenase 12, expressed protein; oxidoreductase, short chain dehydrogenase/reductase family
S8_11,774,122	LOC_Os08g19650, LOC_Os08g19670, LOC_Os08g19680	Homeobox protein knotted-1; expressed protein; expressed protein
S9_4,933,781	LOC_Os09g09220, LOC_Os09g09210	Protein kinase domain containing protein, expressed; expressed protein
S10_14,320,467	LOC_Os10g27170, LOC_Os10g27174, LOC_Os10g27180, LOC_Os10g27160	Calmodulin-binding protein; 40S ribosomal protein S28; expressed protein; hypothetical protein
S10_16,385,834	LOC_Os10g31240, LOC_Os10g31250	Plant protein of unknown function domain containing protein; expressed protein
S11_7,438,223	LOC_Os11g13570, LOC_Os11g13540, LOC_Os11g13560, LOC_Os11g13580	Gibberellin receptor GID1L2; serpin domain containing protein, putative, expressed; serpin domain containing protein, putative, expressed; expressed protein
S4_30,721,851	LOC_Os04g51809, LOC_Os04g51820	Expressed protein; OsHKT1;1—Na + transporter, expressed
S7_24,282,724	LOC_Os07g40520	Formin-like protein 20, putative, expressed
S6_24,807,445	LOC_Os06g41380, LOC_Os06g41384, LOC_Os06g41390	Expressed protein; zinc finger C- × 8-C- × 5-C- × 3-H type family protein, expressed; N-terminal asparagine amidohydrolase
S8_5,775,398	LOC_Os08g09990, LOC_Os08g10000	Expressed protein; expressed protein
S1_40,142,074	LOC_Os01g69070, LOC_Os01g69050, LOC_Os01g69060, LOC_Os01g69080	Auxin efflux carrier component; lysine ketoglutarate reductase trans-splicing related 1; hydrolase, alpha/beta fold family domain containing protein; acyl-desaturase chloroplast precursor
S2_7,048,091	LOC_Os02g13220	F-box family protein
S2_22,216,515	LOC_Os02g36830, LOC_Os02g36820	Cytokinin-O-glucosyltransferase2; expressed protein
S3_7,917,671	LOC_Os03g14590, LOC_Os03g14570, LOC_Os03g14580	Calcium-binding EF hand family protein; expressed protein (conserved gene of known function); expressed protein (in ortholog, conserved gene of known function)
S4_12,374,542	LOC_Os04g21820, LOC_Os04g21830, LOC_Os04g21840, LOC_Os04g21850	OsWAK33—OsWAK receptor-like protein OsWAK-RLP, expressed; hypothetical protein; expressed protein; expressed protein
S4_34,598,600	LOC_Os04g58080, LOC_Os04g58090, LOC_Os04g58100, LOC_Os04g58110	Polygalacturonase inhibitor 3 precursor; harpin-induced protein 1 domain containing protein, Arabidopsis-LEA (LEA) hydroxyproline-rich glycoprotein family/ other ortho NHL25; Expressed Protein; pyruvate kinase
S5_19,130,617	LOC_Os05g32680, LOC_Os05g32660, LOC_Os05g32670, LOC_Os05g32690	Pale Cress Protein (PCP); leucine-rich repeat family protein,; pentatricopeptide repeat-containing protein (ortho-60S ribosomal protein L34); expressed protein
S5_23,720,696	LOC_Os05g40370, LOC_Os05g40384	Expressed protein; cytochrome P450
S5_28,157,471	LOC_Os05g49100	WRKY49
S8_20,423,775	LOC_Os08g32930, LOC_Os08g32920, LOC_Os08g32940	Expressed protein (n ortho-chaperonin like RbcX protein); dynamin-2B; endoglucanase
S8_24,621,885	LOC_Os08g38960, LOC_Os08g38950	Expressed protein (conserved gene of unknown function); EP
S10_111,061	LOC_Os10g01110, LOC_Os10g01134	OsSCP44—Putative Serine Carboxypeptidase homologue; OsSCP46—Putative Serine Carboxypeptidase homologue
S10_19,109,511	LOC_Os10g35750, LOC_Os10g35720, LOC_Os10g35760	Pentatricopeptide; OsGrx_S17—glutaredoxin subgroup II (Pistil); pentatricopeptide
S10_19,238,621	LOC_Os10g35980, LOC_Os10g35990, LOC_Os10g36000	Tetraspanin family protein; DEAD-box ATP-dependent RNA helicase; remorin C-terminal domain containing protein
S1_38,370,584	–	–
S2_2,875,772	LOC_Os02g05820, LOC_Os02g05830, LOC_Os02g05840	Protein kinase domain containing protein; ribulose bisphosphate carboxylase small chain, chloroplast precursor; VIL2 protein
S2_3,873,759	LOC_Os02g07480, LOC_Os02g07490, LOC_Os02g07495	Transglycosylase SLT domain containing protein, expressed; glyceraldehyde-3-phosphate dehydrogenase; expressed protein (pistil)
S4_4,499,266	LOC_Os04g08390, LOC_Os04g08410	Leucine Rich Repeat family protein, expressed (NBS LRR orthologs in brachypodium, Sorghum); ELM2 domain containing protein, putative, expressed
S4_26,914,103	LOC_Os04g45480, LOC_Os04g45460, LOC_Os04g45470, LOC_Os04g45490, LOC_Os04g45500	Heat shock protein STI, putative; cysteine-rich repeat secretory protein precursor; vacuolar-processing enzyme precursor; elongation factor, putative; expressed protein
S4_31,316,844	LOC_Os04g52630, LOC_Os04g52640, LOC_Os04g52614	Leucine-rich repeat-containing protein kinase family protein; SHR5-receptor-like kinase, putative; SHR5-receptor-like kinase
S4_35,115,087	LOC_Os04g59010, LOC_Os04g59020, LOC_Os04g59040, LOC_Os04g59030	F-box domain containing protein; integral membrane protein; soluble inorganic pyrophosphatase, putative; expressed protein
S5_24,316,574	LOC_Os05g41530, LOC_Os05g41510, LOC_Os05g41520, LOC_Os05g41540, LOC_Os05g41550	ZOS5-11—C2H2 zinc finger protein, expressed; SH2 motif, putative, expressed; zinc finger, C3HC4 type domain containing protein, expressed; bZIP transcription factor domain containing protein, expressed; expressed protein
S11_19,062,952	LOC_Os11g32260, LOC_Os11g32250, LOC_Os11g32270	Lysosomal alpha-mannosidase precursor, putative, expressed; expressed protein; N-rich protein, putative, expressed

**Fig. 4:**
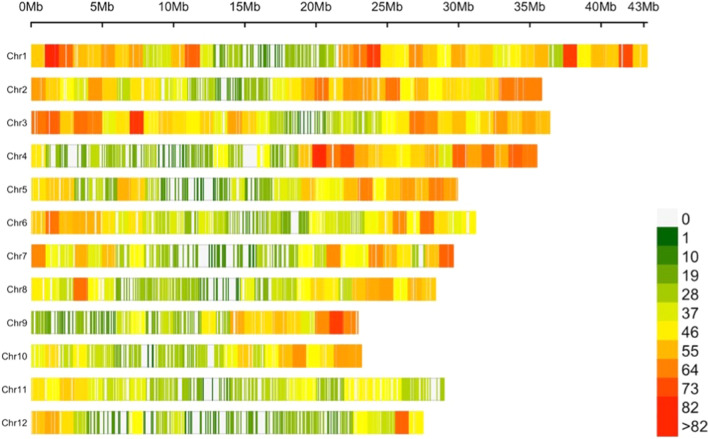
Number of SNPs within 1 Mb window for all the 12 chromosomes

**Fig. 5:**
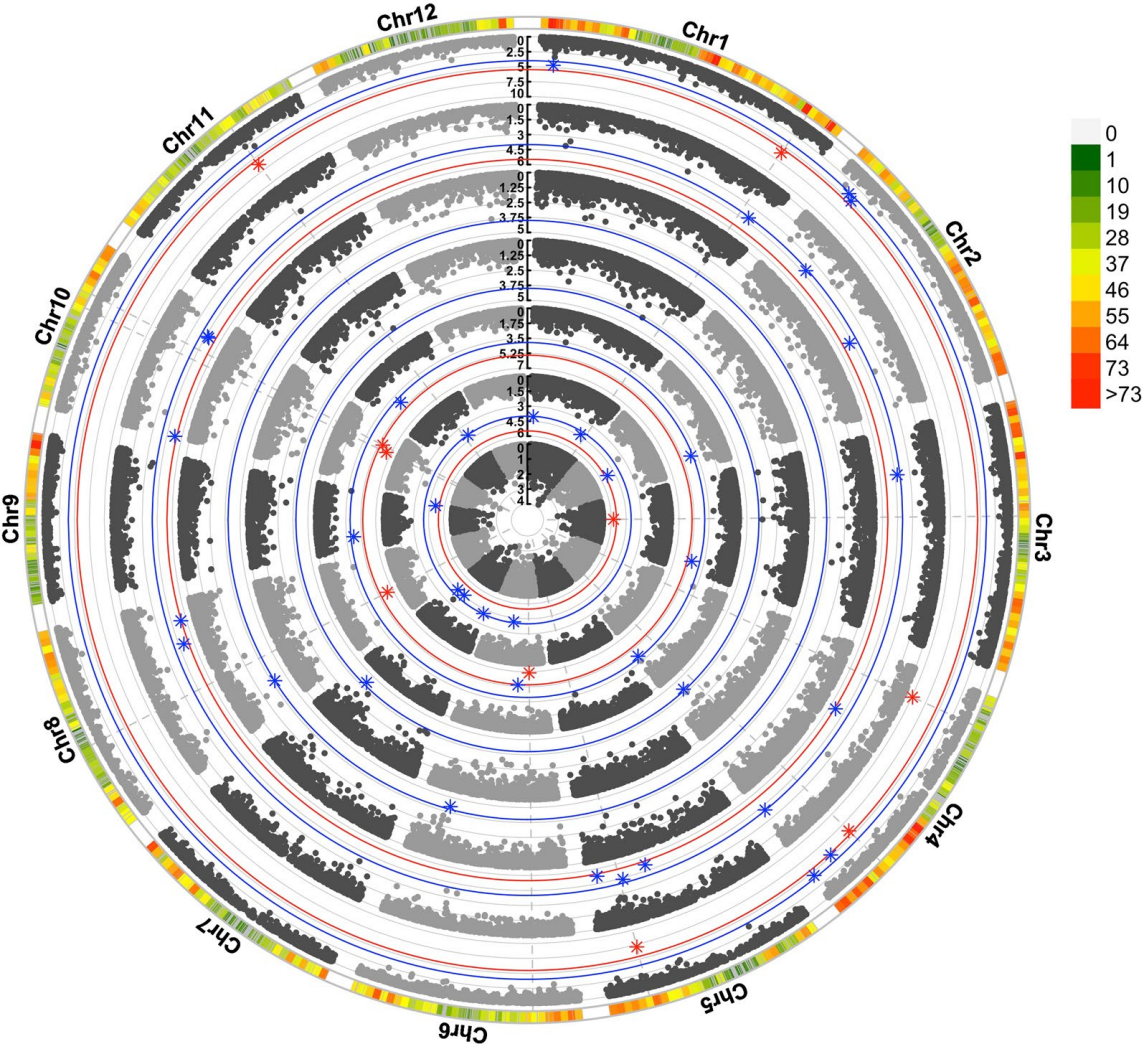
Circular Manhattan plot depicting significant marker trait associations for various traits moving from center towards the periphery plant height, culm thickness, panicle length, number of primary branches per panicle, grain length, grain width and hundred grain weight where red and blue lines represent *p*-value ≤ 1e−6 (Bonferroni threshold) and 1e−4 (threshold in current study), respectively

The distribution of SNP markers chromosome wise is given in Fig. 4. These MTAs were found on all chromosomes, except chromosome 12 as depicted by Manhattan plots presented in Fig. 5. Most of the markers were associated with more than one trait. Markers showing significant association with the trait at *p* ≤ 1e-4 were designated to be strongly associated with the trait and the traits are referred to as primary traits. The association of these strongly associated markers with other traits at lesser stringent *p* value ≤ 0.05 was examined and such traits were designated to be secondary traits. A total of 10 significant SNP associations were obtained each for CT, PL and HGW; 2 each for PB, GL and 14 for GW.

For CT, the significant MTAs altered the value of trait over the mean by a maximum of 7.83% on chromosome 7 while the most significant association, obtained on chromosome 3, altered it by 6.75%. Most of the SNPs associated with CT were also associated with PB and PH at lesser significant *p*-value ≤ 0.05. Sixty percent of the associated MTAs harboured in loci encode proteins like OsCML16—Calmodulin-related calcium sensor protein, terpene synthase, lectin-like receptor kinase 7, chalcone synthase gene, glycosyl hydrolases family 17 protein, peroxisomal multifunctional enzyme type 2 protein. Few loci coding for hsp20/alpha crystallin family protein, nmrA-like family domain containing protein, SMC-related protein MSS2, SMC-related protein MSS2, ubiquitin carboxyl-terminal hydrolase 21 were present in the LD region of these significantly associated SNPs.

The significant MTAs obtained for PL altered the value of trait over mean by a maximum of 8.34% while the most significant association obtained on chromosome 6, altered it by 4.70%. Most of these SNPs were also associated with PH and PB. Ninety percent of significant MTAs harboured in various loci encoding proteins such as helicase domain containing protein, N-rich protein, estradiol 17 beta-dehydrogenase 12, protein kinase domain containing protein, calmodulin-binding protein, gibberellin receptor GIDL2. While a few of them localized in loci encoding expressed proteins of unknown functions, others were in the vicinity of loci encoding tRNA-specific adenosine deaminase, homeobox protein knotted-1.

SNPs most stringently associated with PB were located on chromosomes 4 and 7, S4_30721851 and S7_24282724, respectively. The former SNP altered the trait by a value of 5.56% over the mean value and harboured in LOC_Os04g51809, encoding an expressed protein with highest FPKM values reported in inflorescence. Two other loci, coding for OsHKT1;1—Na + transporter and formin-like protein 20 were present in the LD region of these SNPs. Two significant MTAs obtained for GL on chromosomes, 6 and 8, S6_24807445 and S8_5775398, altered the trait by 1.89% and 1.84%, respectively. SNPs localized in LOC_Os06g41380, LOC_Os08g09990 respectively, both of which encoded expressed protein. Few other loci, coding for zinc finger family protein, N-terminal asparagine amidohydrolase were in the vicinity of these SNPs.

For GW, the most significant MTA, S8_24,621,885, on chromosome 8 altered the trait over the mean value by 3.92% and was localized in LOC_Os08g38960 encoding conserved expressed protein. The SNP was also associated with PB and PH at lesser significant levels. Among all the significant MTAs obtained for GW, S4_12374542, on chromosome 4, had the highest effect, which altered the trait by a value of 4.88% over the mean value. SNP S5_28157471, altered GW by 2.44% over the mean, was also associated with HGW and PH at *p*-value ≤ 0.05. The SNP localized in LOC_Os05g49100, encoding WRKY 49 protein. In total, 10 SNPs significantly associated with GW, localized in loci that encode proteins like auxin efflux carrier component, cytokinin-O-glucosyltransferase, calcium-binding EF hand family protein, Pale Cress Protein (PCP), pentatricopeptide, OsSCP46—Putative Serine Carboxypeptidase homologue, DEAD-box ATP-dependent RNA helicase. Also, some other loci encoding F-box family protein, OsWAK33—OsWAK receptor-like protein OsWAK-RLP, polygalacturonase inhibitor 3 precursor, OsSCP44—Putative Serine Carboxypeptidase homologue, tetraspanin family protein, harpin-induced protein 1 domain containing protein (DS), Arabidopsis-LEA (LEA) hydroxyproline-rich glycoprotein family/other ortho NHL25, leucine-rich repeat family protein, cytochrome P450, dynamin-2B, OsGrx_S17-glutaredoxin subgroup II, lysine ketoglutarate reductase trans-splicing related 1, hydrolase, alpha/beta fold family domain containing protein, pentatricopeptide repeat-containing protein (ortho-60S ribosomal protein L34), pyruvate kinase, hydrolase, alpha/beta fold family domain containing protein, acyl-desaturase, chloroplast precursor were present in the LD region of strongly associated SNPs.

The most significant SNP for HGW, S4_4499266, on chromosome 4 also had the highest effect. Two loci, LOC_Os04g08390 and LOC_Os04g08410, encoding Leucine Rich Repeat family protein, and ELM2 domain containing protein were present within 10 kb region of the SNP. The latter locus had its highest FPKM expression value reported in anthers. Also, this SNP was associated with GL at *p*-value of 0.005, altering it by 2.61% over the mean value. SNP S2_2875772, strongly associated with HGW was also associated with GL and PH, was in LOC_Os02g05830, encoding ribulose bisphosphate carboxylase small chain, chloroplast precursor with highest FPKM values reported in embryo. Another SNP, S2_3873759, present on chromosome 2, was also associated with GL. Three more SNPs, S4_26914103, S4_31316844, S4_35115087, altered the trait by ~ 6%. SNP S4_26914103 were part of LOC_Os04g45480, encoding heat shock protein with highest reported FPKM values in seed. SNP S5_24316574 and S11_19062952, strongly associated with HGW, were localized in LOC_Os05g41530 and LOC_Os11g32260, encoding ZOS5-11-C2H2 zinc finger protein and lysosomal alpha-mannosidase precursor, respectively.

### Allelic Effects

**Fig. 6:**
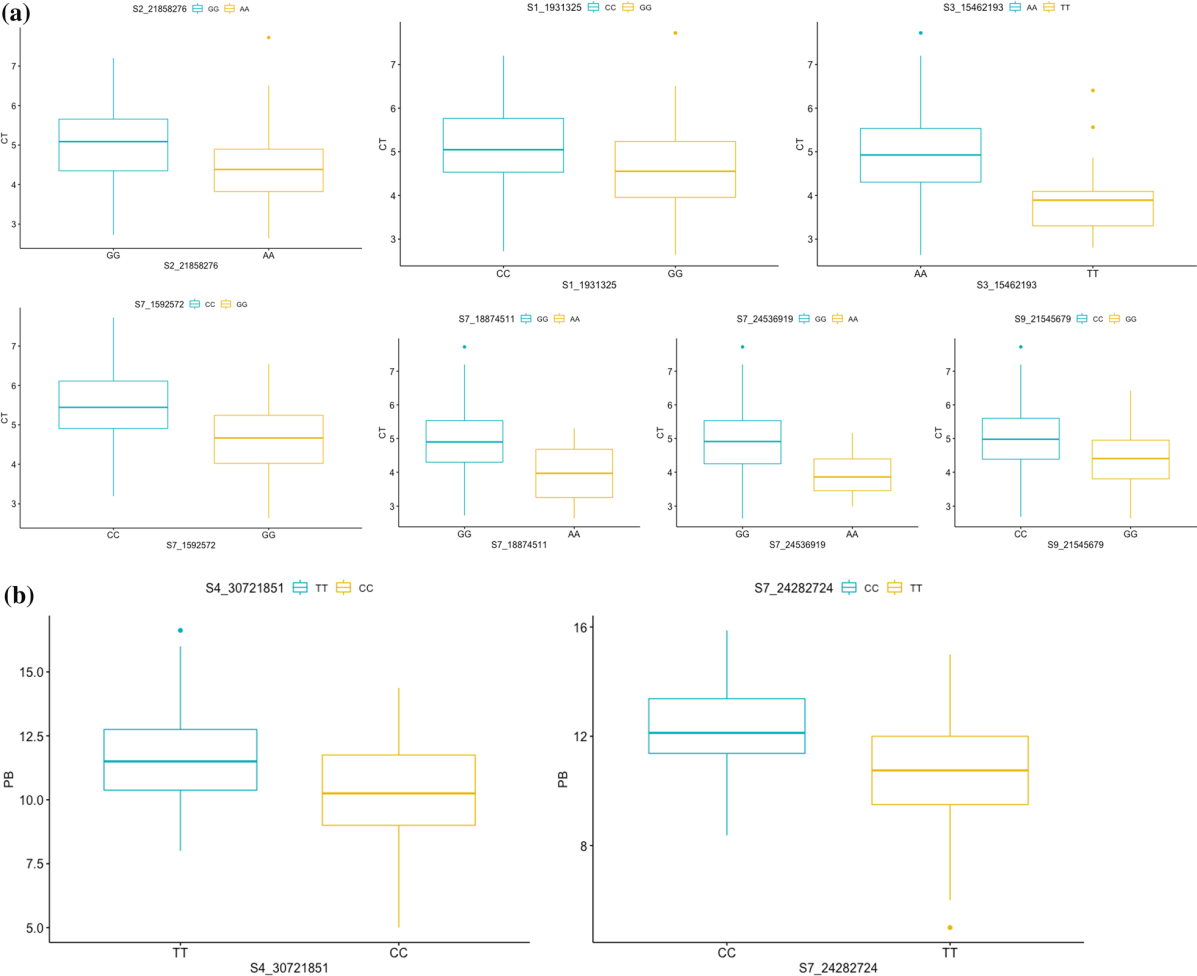

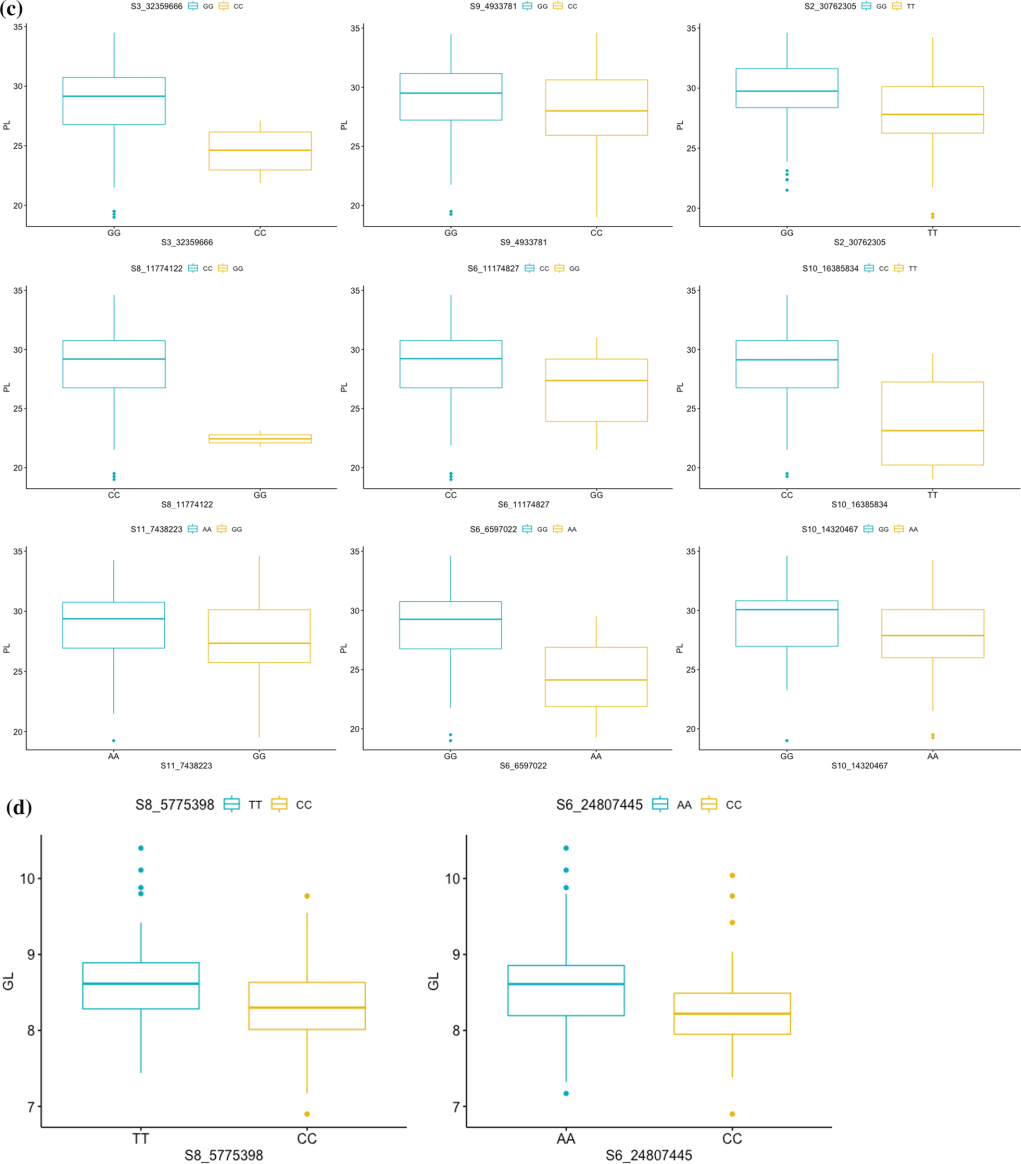

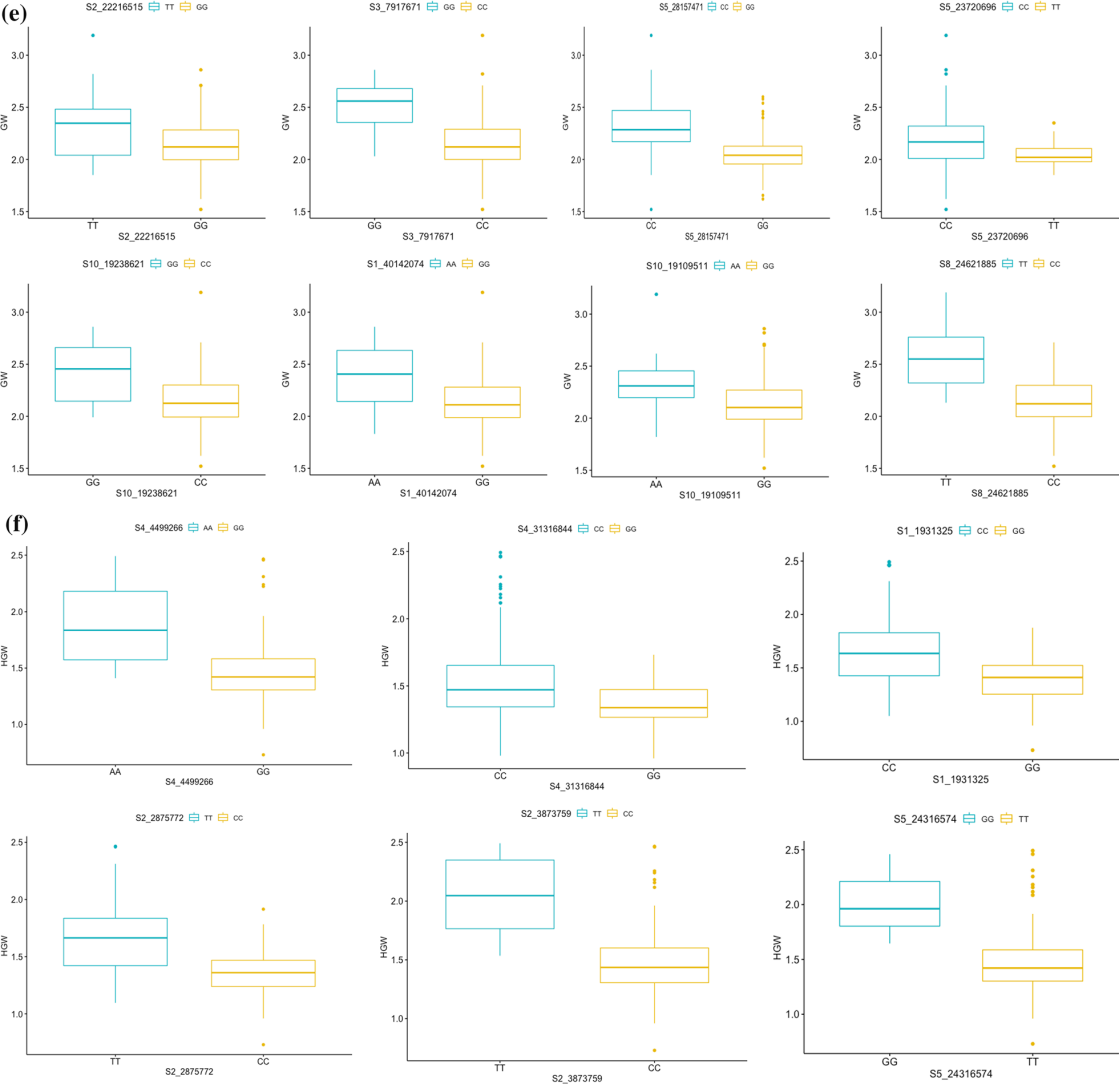
Box and whisker plots depicting significant allelic effects of significant MTAs for all the traits **a** CT **b** PB **c** PL **d** GL **e** GW **f** HGW

The allelic effects of significant MTAs for each trait were evaluated using Kruskal–Wallis test and their chi-square values along with *p*-values have been presented in Table 5 and represented via box-plots in Fig. 6a–f. For all the traits, the differences among alleles were statistically significant at 34 genomic regions. The differences among alleles were significant from breeding point of view as well. For PL, significant MTA with highest effect, S8_11774122, the genotypes with allele CC had on an average 6.3 cm longer panicles than accessions with GG allele. Similarly, for PB, significant SNP with highest effect on chromosome 7, S7_24282724, accessions with genotypes CC had on an average 1.6 more branches relative to accessions with GG genotypes and this difference was statistically significant.

**Table 5:** List of MTAs with significant differences between allelic effects on the basis of Kruskal–Wallis test

Trait	SNP	Chi-square	*p*-value
CT	S1_1931325	19.100	1.24E−05
	S2_21858276	21.327	3.87E−06
	S3_15462193	17.774	2.49E−05
	S7_1592572	31.947	1.24E−05
	S7_18874511	5.212	0.02243
	S7_24536919	10.542	0.001167
	S9_21545679	22.647	1.95E−06
GL	S6_24807445	19.825	8.49E−06
	S8_5775398	15.401	8.69E−05
GW1	S1_40142074	9.623	0.001922
	S2_22216515	4.190	0.04066
	S3_7917671	19.100	1.24E−05
	S10_19238621	6.454	0.01107
	S10_19109511	20.360	6.42E−06
	S5_23720696	4.833	0.02792
	S8_24621885	16.743	4.28E−05
	S5_28157471	50.133	1.44E−12
HGW	S1_1931325	36.661	1.41E−09
	S5_24316574	6.032	0.01405
	S4_31,316,844	4.042	0.04438
	S4_4499266	17.879	2.36E−05
	S2_3873759	16.272	5.49E−05
	S2_2875772	50.715	1.07E−12
PB	S4_30721851	14.919	0.0001122
	S7_24282724	14.063	0.0001768
PL	S2_30762305	12.208	0.0004759
	S3_32359666	11.277	0.0007845
	S6_6597022	15.410	8.65E−05
	S6_11174827	7.097	0.00772
	S8_11774122	5.157	0.02316
	S9_4933781	3.946	0.04699
	S10_14320467	8.116	0.004388
	S10_16385834	7.232	0.007163
	S11_7438223	6.696	0.009661

### Identification of Potential *O. rufipogon* Accessions

The number of *O. rufipogon* accessions possessing superior allelic combinations for CT, PB, PL, HGW, GL and GW, at significant genomic regions were found to be 5, 13, 1, 3, 34 and 1, respectively (Tables 6, 7, 8, 9, 10, 11). However, three accessions, CR100443, IR104777, IR104783 had superior alleles for both GL and HGW. Similarly, CR100459 had superior alleles for PL and PB, IR88788 for GL and CT; and IR103404 for GL and PB. Comparison of *O. rufipogon* genotypes harbouring favourable combination of alleles with *O. sativa* cv. PR114 revealed significant differences phenotypically for CT, PB and GL. However, difference was insignificant for HGW. Significance test could not be performed for PL and GW due to lesser number of accessions having superior allelic combinations. Another comparison between alleles of *O. rufipogon* and an elite *O. sativa* indica cultivar, PR114, at significant genomic regions, revealed superior alleles of wild relative to be absent at 12 loci, implying their introgression into the cultivated germplasm enhance to introduce useful genetic variability (Tables 6, 7, 8, 9, 10, 11).

**Table 6:** The list of *O. rufipogon* accessions possessing combination of superior alleles at significant SNP positions associated with GW where SNPs marked with * represent alleles absent in indica cultivars

	S1_40142074*	S2_22216515	S3_7917671	S5_23720696	S5_28157471	S8_24621885*	S10_19238621*
Superior	AA	GG	GG	CC	CC	TT	GG
IR103308	AA	GG	GG	CC	CC	TT	GG
PR114	GG	GG	NN	CC	CC	CC	CC

**Table 7:** The list of *O. rufipogon* accessions possessing combination of superior alleles at significant SNP positions associated with CT where SNPs marked with * represent alleles absent in indica cultivars

	S1_1931325*	S2_21858276	S3_15462193	S7_1592572	S7_18874511	S7_24536919	S9_21545679
Superior	GG	GG	AA	CC	GG	GG	CC
IR82989	GG	GG	AA	CC	GG	GG	CC
IR83814	GG	GG	AA	CC	GG	GG	CC
IR88788	GG	GG	AA	CC	GG	GG	CC
CR100018A	GG	GG	AA	CC	GG	GG	CC
CR100018	GG	GG	AA	CC	GG	GG	CC
PR114	CC	GG	AA	CC	GG	GG	CC

**Table 8:** The list of *O. rufipogon* accessions possessing combination of superior alleles at significant SNP positions associated with HGW where SNPs marked with * represent alleles absent in indica cultivars

	S1_1931325	S2_2875772	S2_3873759	S4_35115087	S4_4499266*	S5_24316574
Superior	CC	TT	TT	CC	AA	TT
CR100443	CC	TT	TT	TT	AA	TT
IR104777	CC	TT	TT	TT	AA	TT
IR104783	CC	CT	TT	TT	AA	TT
PR114	CC	TT	TT	TT	GG	TT

**Table 9:** The list of *O. rufipogon* accessions possessing combination of superior alleles at significant SNP positions associated with PL where SNPs marked with * represent alleles absent in indica cultivars

	S2_30762305*	S3_32359666	S6_6597022	S6_11174827*	S8_11774122	S9_4933781*	S10_14320467*	S10_16385834	S11_7438223*
Superior	GG	GG	GG	CC	CC	GG	GG	CC	AA
CR100459	GG	GG	GG	CC	CC	GG	GG	NN	AA
PR114	TT	GG	GG	GG	CC	CC	AA	CC	GG

**Table 10:** The list of *O. rufipogon* accessions possessing combination of superior alleles at significant SNP positions associated with PB where SNPs marked with * represent alleles absent in indica cultivars

	S4_30721851	S7_24282724*
Superior	TT	CC
CR100375	TT	CC
CR100449	TT	CC
IR82988	TT	CC
IR81885	TT	CC
IR83810	TT	CC
IR103404	TT	CC
IR89224	TT	CC
IR104404	TT	CC
IR104404A	TT	CC
IR93217	TT	CC
IR104404B	TT	CC
IR105948	TT	CC
CR100459	TT	CC
PR114	TT	TT

**Table 11:** The list of *O. rufipogon* accessions possessing combination of superior alleles at significant SNP positions associated with GL where SNPs marked with * represent alleles absent in indica cultivars

	S6_24807445	S8_5775398*
Superior	AA	TT
CR100013A	AA	TT
CR100443	AA	TT
CR100455	AA	TT
IR100923	AA	TT
IR81801	AA	TT
IR83813	AA	TT
IR103404	AA	TT
IR88788	AA	TT
IR93120	AA	TT
IR104404C	AA	TT
IR100597	AA	TT
IR100678	AA	TT
IR103850	AA	TT
IR104425	AA	TT
IR104641	AA	TT
IR104746	AA	TT
IR104766	AA	TT
IR104777	AA	TT
IR104783	AA	TT
IR104796	AA	TT
IR104821	AA	TT
IR104821A	AA	TT
IR104873	AA	TT
IR105240	AA	TT
IR105726	AA	TT
IR105491	AA	TT
CR100001	AA	TT
IR113661	AA	TT
CR100216	AA	TT
IR80562	AA	TT
CR100368	AA	TT
CR100465B	AA	TT
IR80660	AA	TT
CR100490	AA	TT
PR114	AA	CC

## Discussion

To broaden the genetic base of cultivated rice, it is important to introgress yield enhancing traits from genetically distinct wild relatives in the background of cultivated rice. *O. rufipogon* has already been identified as an important donor of yield contributing traits. A number of accessions of *O. rufipogon* are being conserved in vitro in many germplasm repositories in the world. However, use of a large number of accessions simultaneously is challenging. Therefore, a core set of accessions needs to be identified for their ability to contribute towards yield and yield component traits. Besides, identification of QTLs governing important yield contributing traits from these accessions will speed up the process of transfer in the background of cultivated rice. In this study, diverse *O. rufipogon* accessions showed wide continuous variation for all the seven traits under study. Moderate to high levels of heritability have been obtained ranging from 0.38–0.80, indicating moderate genetic controls of PL, GL, GW to high genetically regulated traits like HGW. The phenotypic data analyses of association mapping panel suggested trait values to be to be notably different from *Oryza sativa* cultivar, PR114, suggesting huge scope of improvement for all these traits. While all the accessions were taller than the cultivar, very tall plants are not very much desirable, being prone to lodging. Approximately, 61%, 93%, 43%, 0.01% and 0.03% of *O. rufipogon* accessions had better CT, PL, PB, GL and GW trait values, respectively, than the elite *O. sativa* cultivar. While some of the accessions like IR104777, IR81989, IR100678, IR81802, IR93119 and IR104873 had better grain characteristics both in terms of length and width; one of the accessions, IR83813 from Myanmar in particular had highest GL and lowest GW. Similarly, some of the Thailand accessions, IR104796, IR104775 and IR104792, promising for promoting CT, also had higher grain weight, PH, PB respectively. A few accessions like IR104783 and IR104766 should be involved in breeding programs aimed at improving grain weight. Also, these accessions had higher values of CT, HGW and GL, respectively. Similarly, a Cambodian accession, IR110406, had higher values of both PL and PB and can be utilized for improving better panicle architecture. Therefore, these accessions should be utilized in breeding programs to transfer useful genetic variability into the cultivated germplasm. Understanding the nature of correlation among various traits that affect yield directly or indirectly, will lead to improved selection rate of better germplasm, thus paving path to superior genetic gains in the breeding programs. In the present research, PH was positively correlated to PL, PB, CT and negatively correlated to grain parameters. Zeng et al. (2017) have also demonstrated a positive correlation of PH with yield in rice. Li et al. (2019) have demonstrated that greater values of 1000-grain-weight, plant height, panicle length account for high grain yield in *indica* rice. However, Joshi and Okuno (2010) have demonstrated a positive significant correlation of number of primary branches, plant height, grain width and grain weight with yield in Tartary buckwheat.

### Population Structure Analysis and LD Decay

Different analytical methods/software demonstrated *O. rufipogon* accessions to be poorly differentiated with 74.27% admixtures. Presence of such a large number of admixtures is reflective of large amount of gene movement among various regions. Also, it indicates the outcrossing nature of germplasm as has earlier been documented ranging from 4–55% (Oka 1988), 10–50% (McCouch et al. 2007), 10.20% (Phan et al. 2012) and 35% (McCouch et al. 2016). Presence of large number of admixtures and introgression hybridization obscures genetic structure in the population as has earlier been documented by Cheng et al. (2003) and thus blurs the boundaries amongst sub-populations, if any. Different studies aimed at investigating population structure in *O. rufipogon* have identified different number of sub-populations, viz., four (Zhou et al. 2003), three (Huang et al. 2012), five (Prathepha et al. 2012), six (Kim et al. 2016), three (Singh et al. 2018). Similarly, the level of differentiation estimated by *F*_*st*_ /AMOVA indicated low to high level of differentiation in various studies. A conclusive statement combining past and present study expressing number of sub-populations in *O. rufipogon* seems unjustified as population structure depends on several factors like set of accessions used for study, their geographical origins, different population size, types and number of markers, and method/technique used for predicting structure. LD decayed very rapidly across *O. rufipogon* genome, and the decay rate was calculated to be 10 kb. Studies by Huang et al. (2012) have also demonstrated similar rate of LD decay and have attributed it to thousands of reproductive cycles and thus several years of recombination, leading to higher mapping resolution in association studies as compared to the domesticated populations.

### Genome Wide Association Study in *O. rufipogon*

GWAS has seen an upward trend in plant sciences since the commencement of this millennium, but it has been challenged by the problem of false positives and false negatives, both of which are equally portentous. Where false positives arising due to unaccounted genetic structure and kinship, lead to practical non-usability of GWAS results during their validation and utilization in biparental mapping populations, false negatives accounted by overcompensating corrections caused by multiple testing (Zhang et al. 2016) and strict statistical level (*p*-value) threshold, lead to loss of true rare SNPs. Therefore, ideally an association mapping (AM) panel with minimum genetic structure or accounted genetic structure in models is employed. Due to absence of strong differentiation in the current AM panel, no structure co-variates were used in the GWAS model in the current study. Generally, a test is statistically significant if the *p*-value is smaller than the pre-defined alpha value. Since GWAS is based on hundreds to thousands of multiple comparisons/testings, the average probability of false positives increases dramatically. For choosing an appropriate significance threshold that distinguishes false positives from false negatives, many corrections have been demonstrated. Out of them, Bonferroni correction is too stringent, gives a very conservative *p*-value threshold, resulting in a huge loss of power and leads to loss of true positives and underrate the goal of genome-wide studies. In our case, LD based determination of significance threshold was also too stringent. Therefore, another threshold was defined based on minimum Bayes Factor using the formula mBF =  − e*P*ln(*P*) as documented by Wakefield (2009), Zhang et al. (2019). However, it led to 194 MTAs. Therefore, in order to narrow down the number of significant SNPs for further detailed annotation, significant threshold for current study was determined to be 0.0001. Overall, 47 MTAs were identified on eleven chromosomes, with no associations observed for any trait on chromosome 12. Previous studies have already reported 42.5% of the total significant MTAs obtained in the current analysis, providing positive analytical support for our findings. Had Bonferroni or LD-based criterion been employed for determining *p*-value, the previously reported QTL regions obtained significant by employing mBF-based correction, would not have been determined significant as only 10 and 6 MTAs were found to be significant by employing former corrections. Thus, deciding an appropriate threshold for GWAS is one of the determining factors of success of GWAS besides accurate phenotyping and modelling of covariates in the model.

Of the 47 significant MTAs reported, only a single SNP, SNP S1_1931325, on chromosome 1, was found to be strongly associated with both CT and HGW. The SNP altered the respective traits by a value of 3.5% and 3.4% over their mean values and localized within LOC_Os01g04330, that encodes an expressed protein OsCML16, gene regulated by OsERF48 transcription factor (TF), whose overexpression in roots led to increased grain yield under drought stress (Jung et al. 2017), thus explaining its association with grain weight. About 40% of the significant MTAs associated with CT harbored in already reported QTLs/genes; *bct2b* (Mu et al. 2004), QTLs AQDZ008 and AQDZ009 (Kashiwagi and Ishimaru 2004) and *bct11* (Mu et al. 2004) reported on 2, 6, 7 and 11 chromosomes. The role of NmrA-like domain containing proteins, Lectin like receptor kinases in cell differentiation, cell division, shoot/fiber development has been documented by Reiner et al. (2016) and Zuo et al. (2004) explaining the association of the significant markers with CT. Similarly, the function of glycosyl hydrolase family proteins, a type of cell wall degrading enzyme, in the control of longitudinal and transverse growth has been linked to CT and PH influencing lodging resistance (Pan et al. 2019). Zhou et al. (2016) have established the interaction of *OsRLCK57* with *OsBRI1* (a rice BR receptor) to affect rice panicle branching, explaining association with PB. Most of these MTAs also showed association with PH and PB at *p*-value ≤ 0.05, evident from positive correlation of CT with PB and PH. For PL, 60% of the significant SNP associations obtained in the present study localized in previously identified QTLs/genes *pl2a*, *qPL-3–2*, *qPL-6*, AQCU085 and AQCU149 as reported by Zhuang et al. (1997), Mei et al. (2003), Kobayashi et al. (2003) and Yanamoto et al. (2016). For the novel MTAs, the expression of proteins encoded by the significant novel genomic regions have their highest reported expression values in panicles/seeds, making these regions strong candidates for new genes/QTLs. The role of Calmodulins being regulators of degradation of tapetal cells and pollen development binding proteins (Zhang et al. 2012; Yu et al. 2016) and GIBBERELLIN INSENSITIVE DWARF (GIDs) in GA perception followed by GA triggered actions (Shimada et al. 2008) like regulation of cell elongation and plant height (Thomas et al. 2005), corroborate the role of this region in regulation of panicle length. A single novel strong association was obtained each for PB and GL, on chromosomes 7 and 8, respectively. Besides this, the other two significant SNPs strongly associated with PB and GL obtained on chromosomes 4 and 6, respectively, have been documented as *qPRB-4a* and *qGL-6* by Teng et al. (2002) and Li et al. (2003). The SNP on chromosome 7, significantly associated with **PB and *PH, localized in locus coding for formin like proteins, reported to play a role in polar pollen cell growth and overexpression leading to broadening of pollen tubes, polar growth changes (Cheung and Wu 2004).

Of all the 14 MTAs reported for GW, SNP S1_40,142,074, on chromosome 1, harbored in previously reported QTL *qGW-1* (Wan et al. 2005). Other MTAs localized/harbored in vicinity of loci encoding proteins belonging to diverse families. The novel SNPs strongly associated with GW on chromosome 2 localized in loci coding for F-box family proteins and cytokinin-O-glucosyltransferases. The latter plays a key role in maintaining the adequate levels of active cytokinins (Takei et al. 2001; Sakano et al. 2004; Abe et al. 2007) essential for modulating the expression of cell cycle regulators which facilitate cell division in the endosperm cells, thus leading to improvement in grain filling (Panda et al. 2018) and seed development (Zhang et al. 2009). Similarly, the role of F box proteins in regulating senescence, seed size and grain number has been reported by (Piao et al. 2009). The only SNP strongly associated with GW on chromosome 3 harbored in locus encoding Calcium-binding EF hand family protein, structural component of Calcium-Dependent Protein Kinases (CPKs), reported to be predominantly abundant in panicle, stamen and seed development (Valmonte et al. 2014). Similarly, OsWAK receptor-like proteins, known to play a role in cell expansion (Lally et al. 2001) are reported to be linked to grain yield, panicle characteristics (Zeng et al. 2017). LRR family protein and pentatricopeptide domain containing protein found in the ld block of MTAs obtained on chromosome 5, are known to regulate panicle/grain size (Su et al. 2012) and plant embryogenesis (Saha et al. 2007), respectively. Furthermore, cytochrome P450s, such as CYP701A8 and CYP714B in rice (Wang et al. 2012; Magome et al. 2013), are considered to play an important role in gibberellin metabolic pathways and biosynthesis of brassinosteroids, known to regulate grain size regulation in rice, including GS5 (Li et al. 2011), *GW5*/*qSW5* (Wan et al. 2008; Weng et al. 2008; Liu et al. 2017). Studies by Hong et al. (2002, 2003) have demonstrated defects in BR biosynthesis leading to smaller seeds. Recently, Ponce et al. (2020) identified a putative cytochrome P450 (Cyp/LOC Os05g08850) to be a possible candidate gene for the *qGW5*. Another MTA on chromosome 10, S10_19,109,511, harbored in LOC_Os10g35750 encoded pentatricopeptides, known to regulation of shape, size and weight of rice grains (Wang et al. 2020). Novel SNP association obtained for GW on chromosome 8 localized in RbcX protein, chaperone involved in biogenesis of Rubisco (Kolesinski et al. 2013), enzyme which fixes inorganic carbon into organic form leading to production of carbohydrates. Another locus in the vicinity coded for dynamin-2B protein, with established role in cellulose biosynthesis as reported by Hirano et al. (2010), Xiong et al. (2010). Also, Li et al. (2017) have demonstrated that mutation in rice dynamin-related gene *OsDRP1E* led to significant alteration in key agronomic traits like plant height, grain weight, panicle length etc. Similarly, another locus found in the LD region, coded for endoglucanase, enzyme responsible for degradation of cellulose, making this LD block to be associated with carbohydrate metabolism and thus grain parameters. The novel MTA for GW obtained on chromosome 10 coded for OsSCP46—Putative serine carboxypeptidase, known to control grain size by regulating grain width and grain filling in GS5, loss of which led to wide and heavy grains owing to dense, slender spikelet epidermal cells as demonstrated by Duan et al. (2017). Another novel MTA, S10_19,238,621, on chromosome 10 localized in DEAD-box ATP-dependent RNA helicase with highest expression in pistil. The other QTLs in the LD block coded for tetraspanins and remorins, known to be involved in floral organ formation (Mani et al. 2015) and grain setting (Gui et al. 2014), making this region to be a strong candidate for grain parameters.

For HGW, 70% of the MTAs obtained in the present study have been previously reported as *gw1.2*/*qTGW1-2*/*gw1.1* (Moncada et al. 2001; Septiningsih et al. 2003; Hittalmani et al. 2002), *QKw2b* (Li et al. 1997), *AQE053*/*gw4* (Xiao et al. 1998; Brondani et al. 2002*), AQEO021* (Redoña and Mackill 1998), *gw5b*/*gw5* (Xiao et al. 1998; Hua et al. 2002), *gw11.1* (Moncada et al. 2001) on chromosomes 1, 2, 4, 5 and 11. Amongst the novel MTAs obtained in present research, S4_35,115,087, on chromosome 4, was located close to loci coding for proteins with F-box domain and soluble inorganic pyrophosphatase enzyme with highest FPKM values reported in seed. Comprehensive analysis of F-box proteins in rice by Jain et al. (2007) suggest their role in floral transition as well as panicle and seed development. Also, loci like OsFBK12 and LARGER PANICLE as reported by Chen et al. (2013) and Li et al. (2011), code for F-box proteins have been reported to regulate seed size, grain number and panicle size, grain weight, grain number, primary branches, respectively, making this region likely to be associated with a novel grain weight region.

### Potential *O. rufipogon* Accessions

The identification and utilization of *O. rufipogon* accessions possessing superior allele combinations at genomic regions significantly associated with trait of interest is one of the promising strategies to introgress useful genetic variability in cultivated gene pool. Thus, identification of 51 *O. rufipogon* accessions possessing superior alleles would enhance the speed of rice breeding operations. Comparison between alleles of *O. rufipogon* and an elite *O. sativa indica* cultivar, PR114, at 34 significant genomic regions revealed superior alleles of wild relative to be absent at 12 loci, implying that despite excessive utilization of *O. rufipogon* in breeding programs, there is still untapped genetic diversity in the progenitor whose introgression in cultivated rice would substantially increase genetic gains.

## Conclusions

Identification of genetic factors underlying agronomically important traits is critical to meet the world's growing demand for high crop yields. Abundant phenotypic variation in wild *O. rufipogon* germplasm coupled with minimum population structure, made this germplasm an ideal panel for conducting association mapping studies. GWAS revealed a total of 47 significant MTAs, out of which 19 were part of previously documented gens/QTLs, providing a positive analytic proof of our study. In-depth genome annotation in the LD region of significant MTAs identified putative candidate genes belonging to F-box proteins, Lectin like receptor kinases, glycosyl hydrolases, Calmodulins, GIDs, formin like proteins, cytokinin-O-glucosyltransferases, OsWAK receptor-like proteins, Cytochrome P450, pentatricopeptides and putative serine carboxypeptidase. The role of majority of the identified putative candidate genes could be established with the trait of interest using previous literature. Validation of the putative candidate genes would contribute to their use in rice breeding programs, broadening the genetic base of cultivated rice, thus making the crop more resilient. Furthermore, genotypes chosen on the basis of improved phenotypic performance along with superior combination of alleles can be directly incorporated into breeding programme to generate pre-breeding material, which will serve as a valuable germplasm resource for rice breeding.

## Methods

### Plant Material and Phenotyping

School of Agricultural Biotechnology, Punjab Agricultural University, Ludhiana is maintaining a large set of wild species accessions belonging to different genomes of rice through clones or seeds. These accessions were originally procured from International Rice Research Institute, Philippines and Central Rice Research Institute, Cuttack. In the present study, a set of 346 accessions of *O. rufipogon* was investigated. The detailed information of these accessions is provided in the Additional file 1: Table S1.

Phenotypic data was collected in replications from 2014–2016 years for seven different traits, namely, plant height (PH), culm thickness (CT), panicle length (PL), number of primary branches per panicle (PB), grain length (GL), grain width (GW) and hundred grain weight (HGW). Data for HGW was recorded in all the three years, while all the other traits were recorded in two years, 2014 and 2015. Briefly, PH and PL was recorded from two different plants and four panicles per accession. The culm thickness was measured from four and six plants respectively with a Vernier caliper. The number of primary branches were counted manually from four panicles. Grains were dehulled and grain parameters; GL and GW, were recorded for 10 grains/accession with grain analyzer. Grain weight was recorded for hundred grains with an electronic weighing balance.

### Statistical Analysis of Phenotypic Data

The phenotypic data was statistically analyzed in R version 3.4. Distribution of averaged phenotypic data was checked by plotting histogram using hist function and by Shapiro–Wilk test. Statistical analysis of phenotypic data was done in R using lme4 package (Bates et al. 2015). For each trait, components of phenotypic variance were estimated from analysis of variance using restricted maximum likelihood methods. The linear mixed effects, lmer function, in lme4 package (Bates et al. 2015) was used to estimate variance components. All the effects were treated as random and broad sense heritability (H^2^) on a line mean basis was calculated.

### DNA Isolation and Genotyping

Large scale DNA was isolated from each accession from 10-day old leaves using Cetyltrimethyl ammonium bromide (CTAB) method (Doyle and Doyle 1987). DNA quality was accessed on 0.8% agarose gel electrophoresis and genomic DNA was quantified using Thermo Scientific NanoDrop™ 8000 spectrophotometer, followed by its normalization to 100 ng μl^−1^. Thereafter, the samples were sent to Genomic Diversity Facility, Cornell University, NY, USA for Genotyping by Sequencing (GBS). Restriction enzyme *ApeKI* was used to generate GBS library.

GBS data was analyzed with the reference-based ‘discovery’ pipeline described in TASSEL 3.0 documentation and in Glaubitz et al. (2014). The vcf file generated after the discovery pipeline, was indexed for use with bwa version 0.7.8-r455. After alignment, file was filtered for the minor allele frequency (maf) > 0.01 and missing data per site < 90% using VCFtools version v0.1.12a. Further filtration was done in unix and R to remove all monomorphic and multi-allelic markers. Also, accessions with missing data points more than 10% were removed to obtain final SNP data file for further analysis.

### Population Structure and Linkage Disequilibrium Analysis

Principal Component Analysis (PCA) and StrAuto program was used to investigate population structure of 346 *O. rufipogon* accessions. Before PCA, the missing data was imputed using A.mat function of rrBLUP package (Endelman 2011). PCA was done on imputed dataset using prcomp function, based on Singular Value Decomposition method. Strauto program (Chhatre and Emerson 2017), based on Structure V2.3.4 software model-based clustering program, was used to infer the population structure. The input file for running STRUCTURE was prepared using PGDSpider (Lischer and Excoffier 2012). The length of burn-in period and number of Monte Carlo Markov Chain (MCMC) replicates after burn-in were set to 100,000 each. The dataset was analyzed for K values ranging from 1–10 with 10 replications/K value. Admixture model-based approach was used to infer the population structure. The best K was determined by Structure Harvester (Earl and vonHoldt 2012) based on Evanno method (Evanno et al. 2005). The outcome of STRUCTURE was plotted with Pophelper package (Francis 2017) in R. Other widely studied parameters for assessing genetic diversity like fixation index (*F*_*st*_*)* and AMOVA were calculated by stamppFst function of StAMPP package (Pembleton et al. 2013) and Poppr package in R (Kamvar et al. 2014), respectively. The stamppFst function of StAMPP package calculates pairwise *F*_*st*_ values along with confidence intervals and *p*-values between populations according to the method proposed by Wright (1949) and updated by Weir and Cockerham (1984). The number of bootstraps was set to 100. LD decay was calculated using PopLDdecay program in unix and was plotted in R using a customized script.

### Genome Wide Association Study

Genome-wide association study (GWAS) was carried out for seven traits, namely, PH, CT, PB, PL, GL, GW, HGW in R using GAPIT 3 (Wang et al. 2020) using a tagged set of 15, 083 SNPs (Additional files 2 and 3: Tables S2 and S3). SNP tagging was done in R using hclust2 function. PCA, STRUCTURE analysis and BIC values indicated absence of genetic structure in the panel, therefore, no covariates were included in the model to correct for population structure. FarmCPU calculated kinship, based on FaSTLMM algorithm, was considered while estimating associations in order to prevent false positives, arising due to population structure. Determining an optimum threshold that determines the significance of a genomic region with trait of interest is of utmost importance to minimize both Type I and Type II errors. Therefore, various corrections such as Bonferroni-correction, LD-based correction and minimum Bayesian approaches were tried and compared. Bonferroni correction was calculated using the formula alpha/n; where alpha = 0.05 and n = 15,083. LD based approach determines effective number of independent tests as LD bins calculated by Reference genome size (390 MB)/Average LD extent (10 Kb). Considering the experiment wide probability of Type-I error to be 0.05, LD-based correction was calculated as documented by Zhang et al. (2015). Minimum Bayes Factor was calculated using the formula e*P*lnP as documented by Goodman (2001) and Zhang et al. (2019). GWAS results were assessed by studying the Quantile–Quantile plots (QQ plots), Manhattan plots and association tables for each trait. The allelic effects were determined for the strongly associated markers by depicting phenotype data for alleles as box plots and using the Kruskal–Wallis test to see if the alleles differ significantly for the associated traits.

## Supplementary Information

**Additional file 1:** The detailed information of *Oryza rufipogon* accessions investigated in current study along with their countries of origin.

**Additional file 2:** Genotypic data of studied 346 accessions.

**Additional file 3:** Map positions of genotypic dataset.

## Data Availability

The genotypic dataset used in current study has been provided as supplementary information. The material used in the study can be requested to the corresponding author.

## Abbreviations

PB: Number of primary branches per panicle
PH: Plant height
PL: Panicle length
CT: Culm thickness
GL: Grain length
GW: Grain width
HGW: Hundred grain weight
GBS: Genotyping by sequencing
GWAS: Genome Wide Association Study (GWAS)
MTA: Marker Trait Associations
mBF: Minimum Bayes factor
LD: Linkage disequilibrium
MAF: Minor allele frequency
PCA: Principal component analysis
QTL: Quantitative trait locus
SNP: Single nucleotide polymorphism
